# Characterization of a (2*R*,3*R*)-2,3-Butanediol Dehydrogenase from *Rhodococcus erythropolis* WZ010

**DOI:** 10.3390/molecules20047156

**Published:** 2015-04-20

**Authors:** Meilan Yu, Meijuan Huang, Qingqing Song, Jianzhong Shao, Xiangxian Ying

**Affiliations:** 1 Engineering Research Center for Eco-Dyeing & Finishing of Textiles, Ministry of Education, Zhejiang Sci-Tech University, Hangzhou 310018, China; E-Mail: meilanyu@zstu.edu.cn; 2 College of Biological and Environmental Engineering, Zhejiang University of Technology, Hangzhou 310014, China; E-Mails: HuangMJroline@163.com (M.H.); Songqqbeauty@163.com (Q.S.)

**Keywords:** (2*R*,3*R*)-2,3-butanediol dehydrogenase, *Rhodococcus erythropolis* WZ010, diacetyl, acetoin, asymmetric reduction

## Abstract

The gene encoding a (2*R*,3*R*)-2,3-butanediol dehydrogenase from *Rhodococcus erythropolis* WZ010 (ReBDH) was over-expressed in *Escherichia coli* and the resulting recombinant ReBDH was successfully purified by Ni-affinity chromatography. The purified ReBDH in the native form was found to exist as a monomer with a calculated subunit size of 37180, belonging to the family of the zinc-containing alcohol dehydrogenases. The enzyme was NAD(H)-specific and its optimal activity for acetoin reduction was observed at pH 6.5 and 55 °C. The optimal pH and temperature for 2,3-butanediol oxidation were pH 10 and 45 °C, respectively. The enzyme activity was inhibited by ethylenediaminetetraacetic acid (EDTA) or metal ions Al^3+^, Zn^2+^, Fe^2+^, Cu^2+^ and Ag^+^, while the addition of 10% (*v*/*v*) dimethyl sulfoxide (DMSO) in the reaction mixture increased the activity by 161.2%. Kinetic parameters of the enzyme showed lower *K*_m_ values and higher catalytic efficiency for diacetyl and NADH in comparison to those for (2*R*,3*R*)-2,3-butanediol and NAD^+^. The activity of acetoin reduction was 7.7 times higher than that of (2*R*,3*R*)-2,3-butanediol oxidation when ReBDH was assayed at pH 7.0, suggesting that ReBDH-catalyzed reaction *in vivo* might favor (2*R*,3*R*)-2,3-butanediol formation rather than (2*R*,3*R*)-2,3-butanediol oxidation. The enzyme displayed absolute stereospecificity in the reduction of diacetyl to (2*R*,3*R*)-2,3-butanediol via (*R*)-acetoin, demonstrating its potential application on the synthesis of (*R*)-chiral alcohols.

## 1. Introduction

As a key enzyme in the microbial production of 2,3-butanediol, 2,3-butanediol dehydrogenase (also known as acetoin/diacetyl reductase) can reduce diacetyl to acetoin and then to 2,3-butanediol that has three stereoisomeric forms: *meso*-2,3-butanediol, (2*R*,3*R*)-2,3-butanediol, and (2*S*,3*S*)-2,3-butanediol [1,2]. Optically pure 2,3-butanediol can be used as valuable building block in asymmetric synthesis of chiral compounds containing two vicinal stereogenic centers [3]. However, native microorganisms are usually unable to produce optically pure 2,3-butanediol stereoisomer [4], which is partially attributed to the *in vivo* coincidence of both (2*R*,3*R*)-2,3-butanediol dehydrogenase and (2*S*,3*S*)-2,3-butanediol dehydrogenase. The pair of genes encoding of (2*R*,3*R*)-2,3-butanediol dehydrogenase and (2*S*,3*S*)-2,3-butanediol dehydrogenase have been confirmed in various strains, e.g., *Raoultella terrigena*, *Klebsiella pneumoniae*, *Bacillus cereus*, *Paenibacillus polymyxa*, *Pseudomonas putida*, and *Corynebacterium glutamicum* [5]. (2*R*,3*R*)-2,3-butanediol dehydrogenases usually belong to the family of zinc-containing alcohol dehydrogenases [5,6,7,8], whereas (2*S*,3*S*)-2,3-butanediol dehydrogenases are commonly clustered in the family of short-chain dehydrogenases/reductases [1,3,9,10,11,12]. Strategies have been developed to overcome the difficulties in the direct fermentation of pure 2,3-butanediol stereoisomer, e.g., constructing the whole cell biocatalysts over-expressing stereospecific 2,3-butanediol dehydrogenase, and constructing metabolic pathways using the emerging synthetic biology tools [13]. Although the application of the novel processes have resulted in high-level production of 2,3-butanediol stereoisomers, the optically pure enantiomers of 100% purity is still difficult to be obtained [14,15,16]. Thus, the discovery and characterization of absolutely stereospecific 2,3-butanediol dehydrogenase would be fundamental for its application on the production of pure 2,3-butanediol stereoisomer.

The *Rhodococcus erythropolis* strains have been proved to be a versatile biocatalyst for stereoselective reduction of ketones to chiral alcohols including d-(−)-pantoyl lactone, (*S*)-3-quinuclidinol and (*S*)-1-phenylethanol [17,18,19]. *R. erythropolis* also serves as a good resource of chiral alcohol dehydrogenases. Up to date, a large set of (*S*)-stereospecific alcohol dehydrogenases have been characterized from *R. erythropolis*, e.g., amino alcohol dehydrogenases from *R. erythropolis* MAK154 and *R. erythropolis* BCRC 10909, (2*S*,3*S*)-2,3-butanediol dehydrogenase from *R. erythropolis* WZ010, L-pantoyl lactone dehydrogenase from *R. erythropolis* AKU2103, secondary alcohol dehydrogenases from *R. erythropolis* DSM 43297 and *R. erythropolis* DSM 44534 [1,20,21,22,23,24,25]. The medium-chain alcohol dehydrogenase characterized from *R. erythropolis* ATCC 4277 was the only enzyme displaying (*R*)-enantioselectivity in asymmetric reduction of ketones [26].

We previously reported the characterization of a (*S*)-stereospecific acetoin/diacetyl reductase from *R. erythropolis* WZ010 and its application on the production of (2*S*,3*S*)-2,3-butanediol [1]. This study describes heterologous expression and characterization of a (2*R*,3*R*)-2,3-butanediol dehydrogenase from *R. erythropolis* WZ010 (ReBDH). The NAD^+^-specific enzyme was strictly (*R*)-enantioselective in ketone reduction and its physiological role was proposed to be involved in the formation of (2*R*,3*R*)-2,3-butanediol.

## 2. Results and Discussion

### 2.1. Over-Expression and Purification of ReBDH

**Figure 1:**
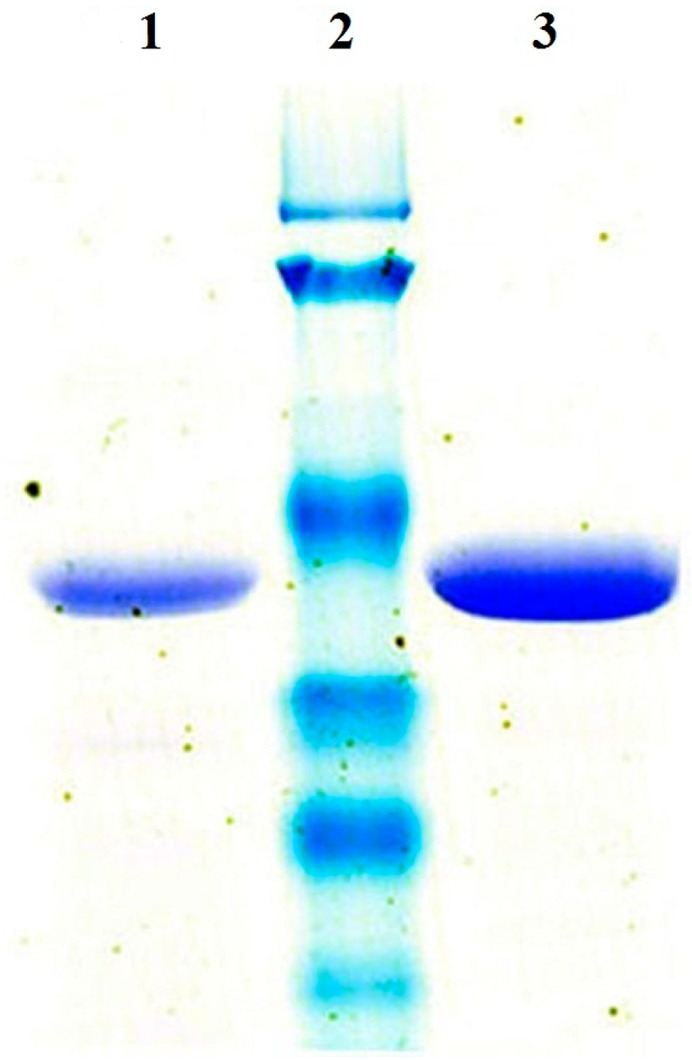
SDS-PAGE analysis of the purified ReBDH with or without N-terminal His-tag. Lane 1, the purified ReBDH without N-terminal His-tag; lane 2, molecular weight marker (from top to bottom: 120, 85, 50, 35, 25, and 20 kDa); lane 3, the purified ReBDH with N-terminal His-tag.

The strain *R. erythropolis* WZ010 was able to produce (2*S*,3*S*)-2,3-butanediol in its alcoholic fermentation [1]. However, trance amounts of (2*R*,3*R*)-2,3-butanediol (around 0.2 mM) were also detected when diacetyl was supplemented as the precursor into the fermentation medium, indicating the possible presence of a gene encoding a (2*R*,3*R*)-2,3-butanediol dehydrogenase in the genome of *R. erythropolis* WZ010. According to the genome sequence of *R. erythropolis* PR4 [27], the gene *rebdh* encoding a putative medium-chain (2*R*,3*R*)-2,3-butanediol dehydrogenase was PCR-amplified from the genomic DNA of *R. erythropolis* WZ010. The gene *rebdh* was subsequently over-expressed in *Escherichia coli* cells using the pEASY-E1 expression vector and the resulting recombinant ReBDH with the N-terminal His-tag was purified to homogeneity by nickel affinity chromatography. The 1056-bp PCR product encoded 351 amino acid residues with a deduced molecular mass of 37180, whereas the purified enzyme migrated as a single band with a size of 44 ± 1 kDa on sodium dodecyl sulfate polyacrylamide gel electrophoresis (SDS-PAGE) (Figure 1). To determine the effect of the N-terminal His-tag, the recombinant ReBDH without the N-terminal his-tag was also successfully over-expressed and then purified using a two-step purification procedure: DEAE-sepharose ion-exchange chromatography and phenyl-sepharose hydrophobic chromatography. The subunit size of ReBDH without the N-terminal his-tag was also 44 ± 1 kDa on SDS-PAGE, indicating the discrepancy between the apparent molecular mass and the deduced molecular mass was due to ReBDH itself but not the N-terminal His-tag. When the NADH absorbance at 340 nm was measured at pH 6.5 and 55 °C, the specific activity of ReBDH with the His-tag was 11.3 U/mg in the acetoin reduction, which was nearly identical to that of ReBDH without the His-tag. For the convenience of enzyme preparation, ReBDH with N-terminal His-tag was used for further characterization. Size exclusion chromatography showed that the native molecular mass of ReBDH was 45 ± 2 kDa and thus ReBDH in the native form was presumably a monomer. The monomeric structure of BeBDH was unusual since (2*R*,3*R*)-2,3-butanediol dehydrogenases typically form either dimers or tetramers [7].

### 2.2. Sequence and Structure Analysis of ReBDH

**Figure 2:**
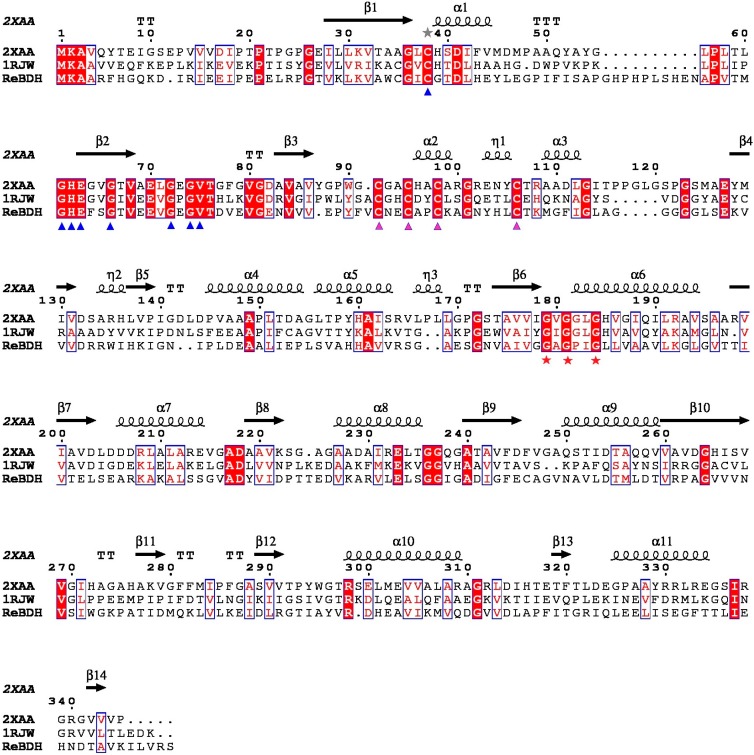
Structure-related sequence alignment between ReBDH and two homologous proteins with known three-dimensional structure. PDB codes for the sequences are as follows: 2XAA, alcohol dehydrogenase from *Rhodococcus ruber* DSM 44541 [28]; 1RJW, alcohol dehydrogenase from *Bacillus stearothermophilus* [29]. Shown above the alignments are elements of the secondary structure of 2XAA. The numbering shown is from 2XAA. Blue triangles, putative catalytic residues; red stars, putative coenzyme binding motif; pink triangles, the residues for the coordination of structural zinc. Strictly conserved residues are highlighted with red boxes.

The nucleotide sequence and the deduced amino acid sequence of ReBDH showed 98.4% and 100% identity with its homologue in *R. erythropolis* PR4 (GenBank accession No. BAH34736, [27]), respectively. A BLAST-P analysis disclosed many putative (2*R*,3*R*)-2,3-butanediol dehydrogenases from the genera *Rhodococcus*, e.g., BDHs from *Rhodococcus* *wratislaviensis* (88% identity; GAF42845), *Rhodococcus opacus* B4 (87% identity; BAH49578), *Rhodococcus* *jostii* RHA1 (87% identity; ABG93446), and *Rhodococcus imtechensis* RKJ300 (87% identity; EID74253). The enzyme ReBDH showed high sequence homology to two NAD^+^-dependent alcohol dehydrogenases with known three-dimensional structure [28,29], and the structure-related sequence alignment revealed that the enzyme was a member of the superfamily of the zinc-containing medium-chain alcohol dehydrogenases (Figure 2). The inductively coupled plasma atomic emission spectrometry (ICP-AES) analyses showed that the purified ReBDH without N-terminal His-tag contained 1.94 ± 0.03 *g*-atoms of zinc per subunit. Therefore, the enzyme could contain 2 *g*-atoms of zinc per subunit in contrast with (2*R*,3*R*)-2,3-butanediol dehydrogenase from *Thermococcus guaymasensis* containing 1 *g*-atom of zinc per subunit [8]. Consistent with the zinc content of ReBDH, the enzyme had all conserved residues for the binding of catalytic and structural zinc ions. The structural zinc binding site was made up of four closely spaced cysteines (Cys_101_, Cys_104_, Cys_107_, and Cys_115_), while the residues (Cys_37_ and His_71_) were critical for the coordination of the catalytic zinc. In addition, the motif of NAD(P)^+^-binding was also identified to be G_179_XG_181_XXG_184_ [30].

### 2.3. Catalytic Properties of ReBDH

**Figure 3:**
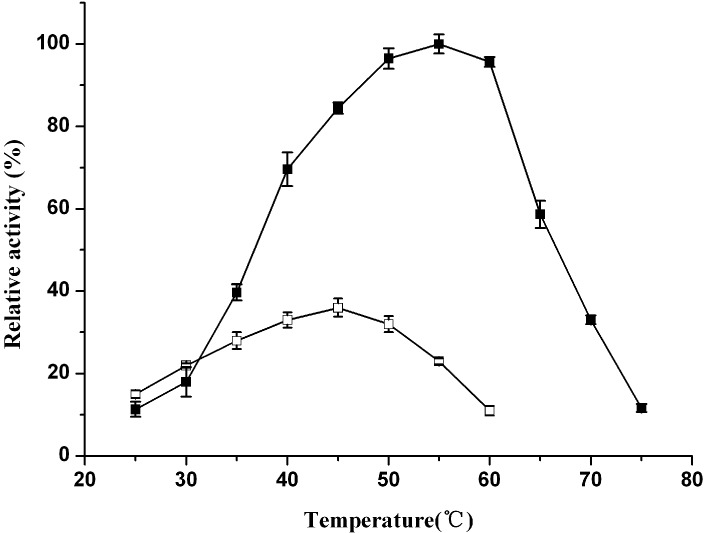
Effect of temperature on the activities of ReBDH. The relative activity of 100% represents 11.3 U/mg for acetoin reduction at 55 °C and pH 6.5. Solid symbols, ketone reduction; empty symbols, alcohol oxidation.

**Figure 4:**
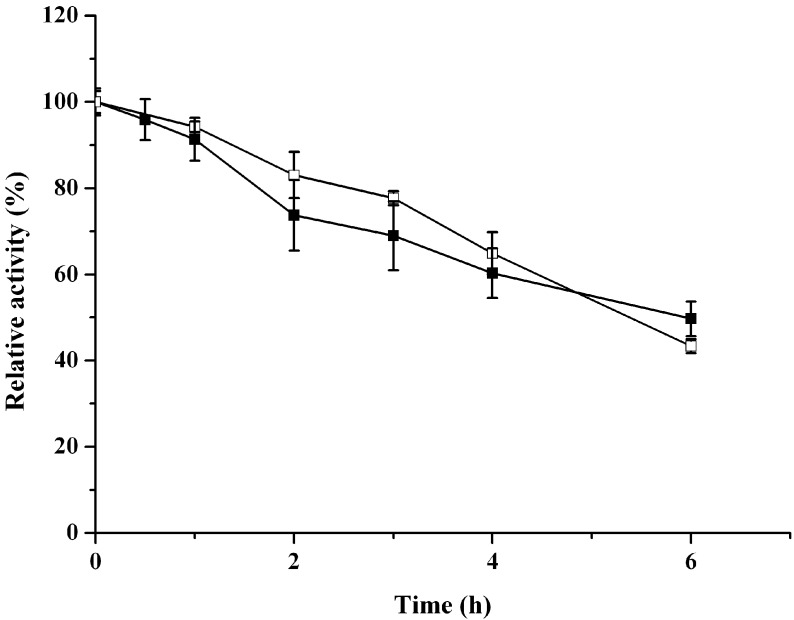
Thermostability of ReBDH. The relative activity of 100% in the alcohol oxidation represents 5.7 U/mg for the oxidation of 2,3-butanediol at 45 °C and pH 10.0. The relative activity of 100% in the ketone reduction represents 11.1 U/mg for acetoin reduction at 55 °C and pH 6.5. Solid symbols, ketone reduction; empty symbols, alcohol oxidation.

The known (2*R*,3*R*)-2,3-butanediol dehydrogenases use NAD(H) and/or NADP(H) as coenzymes [7,8]. With respect to the coenzyme usage, the enzyme ReBDH was strictly NAD^+^-dependent. The enzyme activity was not detectable when NADP(H) was used as coenzyme. The effect of temperature on ReBDH activity was investigated for alcohol oxidation and ketone reduction. Although *R. erythropolis* WZ010 is a mesophilic microorganism, the enzyme in the 2,3-butanediol oxidation was active within a broad temperature range, from 10 to 75 °C. The optimal temperatures of the enzyme were found to be 45 °C for the oxidation of 2,3-butanediol and 55 °C for the reduction of diacetyl (Figure 3). The thermostability of the purified ReBDH was investigated by determining its residual activities when the enzyme samples were incubated at 45 °C (Figure 4). After 6 h incubation at 45 °C, the remaining activities for acetoin reduction and 2,3-butanediol oxidation were 49.7% and 43.5%, respectively. The thermoactivity and thermostability of ReBDH were similar to the properties of alcohol dehydrogenase from *Yokenella* sp. WZY002 that is relatively stable at moderate temperatures and has activity optima at higher temperatures [31]. 

**Figure 5:**
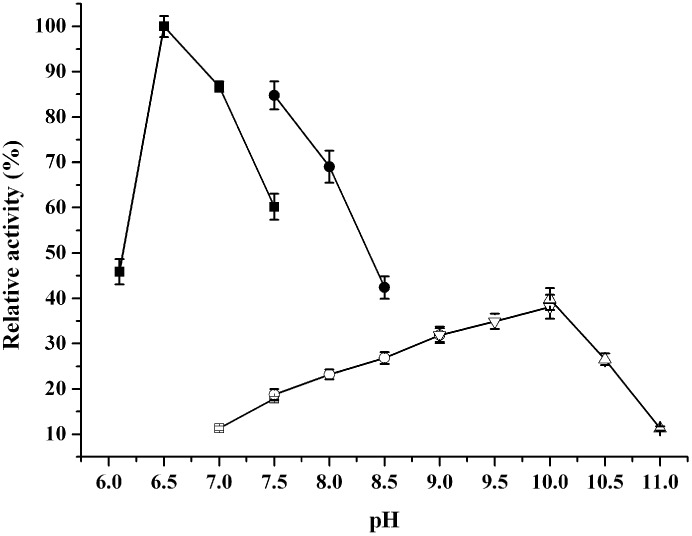
Effect of pH on the activities of ReBDH. The relative activity of 100% represents 11.3 U/mg for acetoin reduction at 55 °C and pH 6.5. Solid symbols, ketone reduction; empty symbols, alcohol oxidation. Buffers used: squares, piperazine-*N*,*N*'-bis(ethanesulfonic acid) (PIPES); circles, Tris-HCl; down-pointing triangles, *N*-cyclohexyl-2-hydroxyl-3-aminopropanesulfonic acid (CAPSO); up-pointing triangles, 3-(cyclohexylamino)-1-propanesulfonic acid (CAPS).

To determine the optimal pHs for both acetoin reduction and 2,3-butanediol reduction, the activity was measured in a pH range of 6.1 to 11.0 (Figure 5). In the alcohol oxidation, the enzyme exhibited the highest activity at pH 10.0. For the reduction of acetoin, >85% of its maximal activity was in the pH range of 6.5 to 7.0, with an optimal pH at 6.5. Similar to the observations on other alcohol dehydrogenases [1,8,31], alcohol oxidation was favored by higher pH since the reaction resulted in the formation of NADH and H^+^. On the other hand, the activity of acetoin reduction was 7.7 times higher than that of (2*R*,3*R*)-2,3-butanediol oxidation when ReBDH was assayed at pH 7.0, revealing that it is more likely to serve as reductase *in vivo*.

The substrate specificity of ReBDH was tested using a set of alcohols, aldehydes, and ketones (Table 1). In the oxidation reaction, the enzyme could oxidize 2,3-butanediol but not acetoin, which is a general property of 2,3-butanediol dehydrogenases [1,8]. Among the tested alcohols, the enzyme exhibited the highest activity for 2,3-butanediol as substrate. Also, the enzyme catalyzed various secondary alcohols including 1-phenyl-1,2-ethanediol, 1,3-butanediol, ethyl 4-chloro-3-hydroxybutyrate, and sodium lactate. In addition, some activity was also observed for 1-butanol, indicating that BeBDH was a primary-secondary alcohol dehydrogenase. In the reductive direction, the activity of ReBDH was found to be the highest on acetoin. Among other tested ketones or aldehydes, diacetyl showed a relatively high activity (69.3%).

Based on the structure analysis, ReBDH belonged to the family of zinc-containing alcohol dehydrogenases. The relative activity in the presence of 1 mM EDTA was reduced to 9.9% (Table 2), indicating that zinc might not be tightly bound to the ReBDH. The cation K^+^ (10 mM) increased the activity by 138.9%, whereas 10 mM cations Na^+^ and Mg^2+^ caused no significant inhibition or activation of ReBDH. The cations (1 mM) including Ca^2+^, Ba^2+^, Mn^2+^ and Co^2+^ decreased the activity by 79.5% to 89.3%. In addition, the cations Al^3+^ and Zn^2+^ decreased the activity to 21.6% and 3.4% of the control enzyme activity, respectively, and the enzyme activity was thoroughly inhibited by Ag^+^, Cu^2+^ and Fe^2+^. ReBDH was not the only case, in which the activity of a zinc-containing enzyme was inhibited by exogenous zinc ion. Similar cases were also observed for the zinc-containing medium-chain alcohol dehydrogenases from *T. guaymasensis*, *Yokenella* sp. strain WZY002, *Acinetobacter* sp. strain M-1 and *Acinetobacter baylyi* ADP1 [8,31,32,33]. The inhibition of the enzyme activity by the mentioned metal ions suggested that sulfhydryl groups of ReBDH might be critical for the enzyme activity [32,33].

**Table 1:** Substrate specificity of ReBDH.

Alcohols	Relative Activity (100%)	Ketones	Relative Activity (100%)
2,3-Butanediol ^a^	100 ^b^ ± 2.7	Acetoin	100 ^c^ ± 4.3
Acetoin	0	Diacetyl	69.3 ± 5.7
(2*R*,3*R*)-2,3-Butanediol	69.5 ± 5.9	2,2,2-Trifluoroacetophenone	28.0 ± 1.8
(2*S*,3*S*)-2,3-Butanediol	0	3-Methyl-2-butenal	16.7 ± 1.3
(*R*)-1-Phenyl-1,2-ethanediol	32.2 ± 2.6	2-Octanone	9.3 ± 1.0
(*S*)-1-Phenyl-1,2-ethanediol	0	2-Hydroxyacetophenone	8.7 ± 0.4
Glycerol	16.3 ± 0.5	Acetophenone	2.5 ± 0.2
1,3-Butanediol	14.7 ± 1.0	4-Hydroxy-2-butanone	0.9 ± 0.2
Ethyl 4-chloro-3-hydroxybutyrate	12.2 ± 0.6		
Sodium lactate	10.2 ± 1.1		
2-Butanol	5.1 ± 0.3		
Ethyl lactate	4.6 ± 0.3		
Isopropanol	3.6 ± 0.4		
1-Butanol	3.1 ± 0.3		
Cyclohexanol	1.5 ± 0.2		
2-Pentanol	1.5 ± 0.2		
2-Octanol	0.5 ± 0.1		

^a^ It consisted of 5.5% (2*S*,3*S*)-2,3-butanediol, 12.9% (2*R*,3*R*)-2,3-butanediol, and 81.9% *meso*-2,3-butanediol; ^b^ The relative activity of 100% represents 5.9 U/mg for 2,3-butanediol oxidation at 45 °C and pH 10.0; ^c^ The relative activity of 100% represents 11.3 U/mg for acetoin reduction at 55 °C and pH 6.5.

**Table 2:** Effect of metal ions and EDTA on the activity of ReBDH.

Compounds	Concentrations (mM)	Relative activity ^a^ (%)
Control	0	100 ^b^ ± 4.3
KCl	10	138.9 ± 5.5
NaCl	10	102.2 ± 1.8
MgCl_2_	10	97.0 ± 4.9
CaCl_2_	1	89.3 ± 5.3
BaCl_2_	1	85.8 ± 2.2
MnCl_2_	1	81.1 ± 1.6
CoCl_2_	1	79.5 ± 3.1
AlCl_3_	1	21.6 ± 3.4
EDTA	1	9.9 ± 0.8
ZnCl_2_	1	3.4 ± 0.3
FeCl_2_	1	0
CuSO_4_	1	0
AgNO_3_	1	0

^a^ The experiments were carried out in triple replicates; ^b^ The relative activity of 100% represents 11.1 U/mg for acetoin reduction at 55 °C and pH 6.5.

Enzymes resistant to organic solvents are of great interest for the purpose of practical biocatalysis. When organic solvent such as methanol, ethanol, acetone, or acetonitrile was added into the assay mixture at a final concentration of 10% (*v*/*v*), the residual activity remained at 2.4%–38.3% of the control enzyme activity (Table 3). Remarkably, when DMSO at a final concentration of 10% or 20% (*v*/*v*) was added into the assay mixture, the activity was increased up to 161% and 122% of the control enzyme activity, respectively. The activity of ReBDH at 30% (*v*/*v*) DMSO still remained 83.8% of the control enzyme activity. Owing to the DMSO tolerance of ReBDH, the use of aqueous solvent such as DMSO would provide the advantages to solubilize more hydrophobic substrates in biocatalysis [34].

**Table 3:** Effect of organic solvents on the activity of ReBDH.

Organic Solvents	Concentrations (%)	Relative Activity (100%)
Control	0	100 ^a^ ± 4.3
DMSO	10	161.2 ± 2.8
	20	122.5 ± 3.4
	30	83.8 ± 3.1
	40	8.4 ± 0.3
Acetone	10	38.3 ± 1.1
Methanol	10	14.0 ± 0.3
Ethanol	10	3.7 ± 0.2
Acetonitrile	10	2.4 ± 0.2

^a^ The relative activity of 100% represents 11.3 U/mg for acetoin reduction at 55 °C and pH 6.5.

### 2.4. Kinetic Parameters of ReBDH

Kinetic parameters of ReBDH in the (2*R*,3*R*)-2,3-butanediol oxidation were determined at pH 6.5 and 55 °C, while the assay conditions determining kinetic parameters of ReBDH in the diacetyl reduction were pH 10.0 and 45 °C. The *K*_m_ value for diacetyl (0.1 mM) was 5.8-fold lower than that for (2*R*,3*R*)-2,3-butanediol (0.58 mM). The specificity constant *K*_cat_/*K*_m_ for diacetyl (61100 s^−1^·M^−1^) was 8.37 times higher than that for (2*R*,3*R*)-2,3-butanediol (7379 s^−1^·M^−1^). The apparent *K*_m_ value for the coenzyme NADH was 13 times lower than that for the coenzyme NAD^+^ (Table 4). The specificity constant *K*_cat_/*K*_m_ for NADH as an electron donor in the diacetyl reduction (71625 s^−1^·M^−1^) was 7.25 times higher than that of the electron acceptor NAD^+^ in the oxidation of corresponding alcohol (9875 s^−1^·M^−1^). 

**Table 4:** Kinetic parameters of ReBDH.

Substrate	Cosubstrate (mM)	Apparent *K*_m_ (mM)	*V*_max_ (U/mg)	*K*_cat_ (s^−1^)	*K*_cat_/*K*_m_ (s^−1^·M^−1^)
(2 *R*,3*R*)-2,3-Butanediol	NAD^+^ (0.64)	0.58 ± 0.05	6.65 ± 0.18	4.28 ± 0.12	7379 ± 860
NAD^+^	(2 *R*,3*R*)-2,3-butanediol (50)	1.04 ± 0.11	15.96 ± 1.07	10.27 ± 0.69	9875 ± 1777
Diacetyl	NADH (0.4)	0.1 ± 0.01	9.49 ± 0.22	6.11 ± 0.14	61100 ± 7650
NADH	Diacetyl (50)	0.08 ± 0.01	8.92 ± 0.12	5.73 ± 0.08	71625 ± 10078

### 2.5. Stereoselectivity of ReBDH

In the alcohol oxidation, ReBDH demonstrated activities on *meso*-2,3-butanediol, (2*R*,3*R*)-2,3-butanediol and (*R*)-1-phenyl-1,2-ethanediol, but not (2*S*,3*S*)-2,3-butanediol and (*R*)-1-phenyl-1,2-ethanediol, suggesting that the enzyme stereoselectively functioned on the (*R*)-hydroxyl group of 2,3-butanediol and 1-phenyl-1,2-ethanediol as substrate (Table 1). The stereoselectivity of ReBDH in the oxidation of 2,3-butanediol stereoisomers was superior to that of (2*R*,3*R*)-2,3-butanediol dehydrogenase from *T. guaymasensis*, which could function on (2*S*,3*S*)-2,3-butanediol [8]. To further approve the stereoselectivity of ReBDH, the ketone reduction and its products were further investigated. In the reduction reaction coupled with formate dehydrogenase-catalyzed coenzyme regeneration, the ReBDH-catalyzed reduction of diacetyl led to optically pure (*R*)-acetoin and (2*R*,3*R*)-2,3-butanediol with 35.7% and 34.2% yields, respectively (Table 5). In the coupled reaction, the concentration of NAD^+^ used was only 0.25 mM and its total turnover number (TTN) was calculated to be 167.6, indicating that the coenzyme regeneration was highly efficient. Moreover, the reduction of racemic (*R*/*S*)-acetoin formed (2*R*,3*R*)-2,3-butanediol and *meso*-2,3-butanediol, which were presumably originated from (*R*)-acetoin and (*S*)-acetoin, respectively, indicating that the reduction of acetoin was also (*R*)-stereoselective.

**Table 5:** Asymmetric reduction of ketones catalyzed by ReBDH.

Substrate (mM)	Conversion (%)	Products	Yield (%)	*e*.*e*. ^a^ (%)
Diacetyl (60)	73.6 ± 4.0	( *R*)-acetoin	35.7 ± 2.2	100 ^b^
		(2 *R*,3*R*)-2,3-butanediol	34.2 ± 1.7	100 ^b^
Racemic acetoin (60)	42.6 ± 3.6	(2 *R*,3*R*)-2,3-butanediol	24.7 ± 2.4	100 ^c^
		*meso*-2,3-butanediol	14 ± 1.1	100 ^c^

^a^*e*.*e*., enantiomeric excess value; ^b^ 100% *e*.*e.* means the amounts of (*S*)-acetoin, *meso*-2,3-butanediol and (2*S*,3*S*)-2,3-butanediol were not detectable; ^c^ 100% *e*.*e.* means that the amount of (2*S*,3*S*)-2,3-butanediol was not detectable.

## 3. Experimental Section

### 3.1. Materials Used

All chemicals were purchased from J&K Chemical Ltd. (Shanghai, China) or Shanghai Jingchun Reagent Co., Ltd. (Shanghai, China). Restriction enzymes were purchased from TaKaRa Bio Inc. (Dalian, China). TransStart^TM^ Taq DNA Polymerase for polymerase chain reaction (PCR) amplification, pEASY-E1 and pEASY-E2 expression vectors were purchased from TransGen Biotech Co., Ltd. (Beijing, China).

### 3.2. Microorganisms and Growth Conditions

The *E. coli* DH5α strain was used as the host for the cloning vectors, and *E. coli* BL21(DE3) was used for the purpose of over-expression. *R. erythropolis* WZ010 used as the donor of the *rebdh* gene had been deposited in the China Center for Type Culture Collection (CCTCC M 2011336). The Luria–Bertani (LB) medium with a NaCl concentration of 5 g/L was routinely used for culturing recombinant *E. coli* and preparing seed inocula of *R. erythropolis* WZ010. All bacteria were cultured at 30 °C, 200 rpm for 24 h, unless stated otherwise. The alcoholic fermentation of *R. erythropolis* WZ010 was carried out in a previously described fermentation medium containing 110 mM glucose [35]. After 12 h of alcoholic fermentation, 2 mM diacetyl was supplemented into the fermentation medium. The end products such as acetoin and 2,3-butanediol were determined using a chiral gas chromatography (GC) method as previously described [1].

### 3.3. Construction of the Expression Plasmids pEASY-E1-rebdh and pEASY-E2-rebdh

The gene of *R. erythropolis* WZ010 encoding the putative ReBDH, named as *rebdh*, was amplified from the genomic DNA using forward and reverse primers rebdhF1 (5'-ATGAAGGCAGCACGGTTC-3') and rebdhR1 (5'-TCACGACCTGACGAGAATCT-3'). The *rebdh* gene was PCR-amplified with the conditions as follows: 94 °C for 4 min for initial denaturalization; 30 cycles of 94 °C for 30 s, 57 °C for 1 min, and 72 °C for 50 s; and 72 °C for 10 min for the final extension. The PCR products were purified and then ligated with the expression vector through the AT ligation strategy, according to the instruction of the pEASY-E1 expression kit (TransGen Biotech Co., Ltd., Beijing, China). The ligated product was subsequently transformed into *E. coli* DH5α competent cells. The recombinant plasmid harboring the *rebdh* gene, designated as pEASY-E1-*rebdh*, was isolated from positive transformers screened through colony PCRs and further verified by DNA sequencing (Sangon Biotech, Shanghai, China). Following the same procedure, the plasmid pEASY-E2-*rebdh* was constructed using the vector pEASY-E2 instead of pEASY-E1.

### 3.4. Expression and Purification of ReBDH with or without N-terminal His-tag

Chemically competent cells *E. coli* BL21(DE3) were transformed with the resulting plasmid pEASY-E1-*rebdh* or pEASY-E2-*rebdh*. The recombinant cells containing pEASY-E1-*rebdh* or pEASY-E2-*rebdh* were grown at 37 °C in the LB medium until the OD_600_ reached up to 0.5, and subsequently induced by adding 1 mM isopropyl β-d-1-thiogalactopyranoside (IPTG). After 16 h of growth 28 °C, *E. coli* cells were harvested by centrifugation and resuspended in 50 mM Tris-HCl buffer (pH 8.0). The cells were disrupted through ultrasonication for 8 min, and the cell lysate was then centrifuged to remove the cell debris, resulting in a clear cell extract. The crude cell extracts were used for the purification of recombinant ReBDHs.

To purify the recombinant ReBDH with N-terminal His-tag from the transformant *E. coli* BL21(DE3)/pEASY-E1-*rebdh*, the obtained supernatant from the crude extract was applied to a Ni-NTA chelating affinity column (Bio-Rad Laboratories, Hercules, CA, USA) equilibrated with the binding buffer (5 mM imidazole and 300 mM NaCl dissolved in 50 mM Tris-HCl, pH 8.0). Unbound proteins were removed by washing with the binding buffer, and the recombinant ReBDH was eluted by applying a stepwise gradient of imidazole concentrations from 10 to 250 mM. Fractions containing ReBDH were eluted with 100 mM imidazole, desalted with 50 mM Tris-HCl (pH 8.0) by ultrafiltration, and then stored at −20 °C for further characterization. 

In the purification of the recombinant ReBDH without N-terminal His-tag from the transformant *E. coli* BL21(DE3)/pEASY-E2-*rebdh*, the cell extract was applied to a 20-ml DEAE-sepharose column, and the enzyme ReBDH was eluted using a linear gradient of 0 to 1 M NaCl in 50 mM Tris-HCl buffer (pH 8.0). The fractions containing ReBDH activity were pooled and applied to a phenyl-sepharose column equilibrated with 50 mM Tris-HCl buffer (pH 8.0) containing 0.8 M (NH_4_)_2_SO_4_. Proteins were eluted with a linear gradient of 0.8 to 0 M (NH_4_)_2_SO_4_. In the phenyl-sepharose hydrophobic chromatography, the recombinant ReBDH without N-terminal His-tag was not eluted out using 50 mM Tris-HCl buffer (pH 8.0) but ultrapure water. After purification, the samples of ReBDH without N-terminal His-tag were washed with 50 mM Tris-HCl (pH 8.0) by ultrafiltration and then stored at −20 °C for further use.

### 3.5. Size Exclusion Chromatography and Structural Analysis

The purity of the purified ReBDHs was verified using SDS-PAGE as described previously [36]. The molecular mass of the enzyme in the native form was determined by a high-performance liquid chromatography on a size-exclusion column WAT011535 (Waters Corporation, Milford, MA, USA). The mobile phase was 50 mM phosphate buffer containing 150 mM NaCl (pH 6.8), and the proteins used for calibration were thyroglobulin (670 kDa), γ-globulin (158 kDa), ovalbumin (44 kDa), myoglobin (17 kDa), and vitamin B12 (1.35 kDa). The homologues of ReBDH were identified by performing BLAST-P searches [37]. The alignment of ReBDH and its close homologues were carried out using the program ClustalW and subsequently visualized using ESPript 3.0 [38,39].

### 3.6. Determination of Zinc Content in ReBDH

The zinc content of ReBDH was determined using ICP-AES (IRIS Intrepid ICP-AES at the Ocean College, Zhejiang University of Technology). The purified ReBDH without N-terminal His-tag was pretreated to wash off nonbinding metals using ultrapure water. The washing procedure was carried out using YM-10 Amicon centrifuge tubes, including five repeats of centrifugation (concentration and refilling of washing buffer). The passthrough solution from the last washing was collected as the control. 100 μL concentrated protein sample containing 0.88 mg ReBDH or the control solution was mixed with 1 mL nitric acid and then digested at 90 °C for 4 h. Prior to ICP-AES analyses, the digested samples were diluted to a final volume of 10 mL using ultrapure water.

### 3.7. Activity Assays of ReBDH

The catalytic activity of ReBDH was measured by following either the reduction of NAD^+^ or the oxidation of NADH at 340 nm (ε_340_ = 6.3 mM^−1^·cm^−1^). Unless otherwise specified, the enzyme assay was carried out in duplicate using the assay mixture (2.5 mL) for alcohol oxidation containing 50 mM 2,3-butanediol and 0.64 mM NAD^+^ at 45 °C in the 50 mM CAPS buffer (pH 10.0). The assay mixture (2.5 mL) for the reduction of ketone/aldehyde contained 50 mM diacetyl and 0.4 mM NADH at 55 °C in the 50 mM PIPES buffer (pH 6.5). The reaction was initiated by the addition of 40 μg of the purified enzyme. One unit of the activity was defined as the amount of enzyme that oxidized or reduced 1 μmol NADH or NAD^+^ per minute. The protein concentrations of all samples were determined using the Bradford reagent (Bio-Rad) with bovine serum albumin as the standard protein [40].

### 3.8. Catalytic Properties of ReBDH

The effect of temperature on the enzyme activity was examined (ranged from 15 to 75 °C) using the 50 mM PIPES (pH 6.5) or the 50 mM CAPS (pH 10.0). To investigate the thermostability of ReBDH, the residual activity was measured by the standard assays for acetoin reduction and 2,3-butanediol oxidation with incubation at 45 °C for appropriate time intervals. The effect of pH on the enzyme activity was determined over a range of pH 6.1 to 11.0 at 45 °C (oxidation) or 55 °C (reduction). The buffers (50 mM) used were PIPES (pH 6.1 to 7.5), Tris-HCl (pH 7.5 to 9.0), CAPSO (pH 9.0 to 10.0), and CAPS (pH 10.0 to 11.0). All the pH values of the buffers used were determined at 25 °C using a Mettler Toledo FE20 FiveEasy pH Meter. With regard to substrate specificity, the activity of ReBDH was tested on various substrates, including primary and secondary alcohols, diols, polyols, and aromatic alcohols or aldehydes and ketones under standard assay conditions.

The effect of cations, EDTA, and organic solvents on enzyme activity was examined by adding each compound at a final concentration of 1 mM (metal ions and EDTA) or 10% (*v*/*v*) (organic solvents) unless stated otherwise. The residual enzyme activity was determined by measuring the reduction of diacetyl in the 50 mM PIPES (pH 6.5), and the control enzyme activity was assayed in the absence of any test compound. 

Enzyme kinetic parameters were determined using different substrates and coenzymes (NAD^+^ or NADH). Various substrate concentrations were used for determining the corresponding activities at the appropriate temperature (55 °C for ketone reduction and 45 °C for alcohol oxidation) when concentrations of corresponding coenzymes were kept constant. Substrates and coenzymes used were NAD^+^ (0, 0.05, 0.1, 0.2, 0.3, 0.4, 0.5, 0.6, 0.7, and 0.8 mM), (2*R*,3*R*)-2,3-butanediol (0, 0.45, 2.3, 5.8, 11.6, 23.2, 34.8, 46.4, 58, and 116 mM), NADH (0, 0.05, 0.1, 0.2, 0.3, 0.4, 0.5, and 0.6 mM), diacetyl (0, 0.45, 2.3, 5.8, 11.6, 23.2, 34.8, 46.4, and 92.8 mM). Apparent values of *K*m and *V*max were calculated by a computer-aided direct fit to the Michaelis–Menten equation using SigmaPlot (Systat Software Inc., San Jose, CA, USA). All the reactions followed Michaelis–Menten-type kinetics.

### 3.9. Asymmetric Reduction of Diacetyl and Acetoin Catalyzed by ReBDH

The enzyme used for *in situ* regeneration of NADH during asymmetric synthesis was the mutant of formate dehydrogenase from *Candida boidinii* ATCC32195 with two amino acid replacements of Csy23Ser and Cys262Ala [41]. The gene encoding the mutant of formate dehydrogenase from *Candida boidinii* ATCC32195 was synthesized and then inserted into *Nde* I and *Xho* I restriction sites of the plasmid pET-30a. The resulting expression plasmid pET-30a-*cbfdh* was transformed into chemically competent cells *E. coli* BL21(DE3). The expression and purification of formate dehydrogenase from *Candida boidinii* ATCC32195 were carried out following the procedures for expression and purification of ReBDH with N-terminal His-tag in Section 3.4.

To determine the stereoselectivity of ReBDH, the asymmetric reduction of diacetyl/acetoin was carried out using formate dehydrogenase-catalyzed coenzyme regeneration. The reduction mixture (2 mL) containing 60 mM diacetyl or racemic acetoin, 120 mM sodium formate as cosubstrate, 0.25 mM NAD^+^, 5 U recombinant formate dehydrogenase from *Candida boidinii* ATCC32195 and 2.5 U ReBDH in the 50 mM PIPES buffer (pH 7.0). The reaction was carried out at 30 °C for 48 h under static condition, unless otherwise specified. The reactants were determined with a Shimadzu GC-2014 gas chromatograph (Shimadzu Corporation, Kyoto, Japan) equipped with a chiral GC column (Varian CP7502, 25 m × 0.25 mm × 0.39 mm) as previously described [1]. The 2 mL reaction mixture was extracted with 1 mL 1-butanol under strong vibration. The reaction mixture after extraction was dehydrated with anhydrous sodium sulfate and then 1 μL dehydrated sample was directly applied onto the injector (250 °C) for GC analyses. The peak areas were quantitated using specific external standards. Retention times of the reactants were listed as follows: 3.25 min for diacetyl, 6.30 min for (*R*)-acetoin, 6.60 min for (*S*)-acetoin, 9.16 min for (2*S*,3*S*)-2,3-butanediol, 9.25 min for (2*R*,3*R*)-2, 3-butanediol, and 9.42 min for *meso*-2,3-butanediol.

### 3.10. Nucleotide Sequence Accession Number

The nucleotide sequence of ReBDH has been submitted to the GenBank database under the accession number of KP868656.

## 4. Conclusions

The strain *R. erythropolis* WZ010 had the capacity to produce both (2*S*,3*S*)-2,3-butanediol and (2*R*,3*R*)-2,3-butanediol, revealing the presence of the gene encoding (2*R*,3*R*)-2,3-butanediol dehydrogenase in its genome. The enzyme ReBDH with monomeric structure shared common structural characteristics with other homologues in the family of medium-chain zinc-containing alcohol dehydrogenases. ReBDH catalyzed the oxidation of 2,3-butanediol to acetoin and the reduction of diacetyl/acetoin to (2*R*,3*R*)-2,3-butanediol. ReBDH had higher catalytic efficiency on NADH and diacetyl (determined at pH 6.5 and 55 °C) than that on NAD^+^ and (2*R*,3*R*)-2,3-butanediol (determined at pH 10.0 and 45 °C). When the assay pH (e.g., pH 7.0) was close to the physiological pH, the activity of ReBDH for acetoin reduction was much higher than that for (2*R*,3*R*)-2,3-butanediol oxidation, clearly indicating that the preference for ReBDH-catalyzed (2*R*,3*R*)-2,3-butanediol oxidation or acetoin reduction was a pH-driven process. Taken together, the results suggested that the physiological role of ReBDH was very likely to be associated with the formation of (2*R*,3*R*)-2,3-butanediol rather than the oxidation of (2*R*,3*R*)-2,3-butanediol.

The enzyme was active within broad ranges of pH and temperature and had remarkable activity in the presence of higher concentration of DMSO, offering greater flexibility in practical biocatalysis. Furthermore, both the oxidation of 2,3-butanediol/1-phenyl-1,2-ethanediol and the reduction of diacetyl/acetoin catalyzed by ReBDH were strictly (*R*)-enantioselective. Besides (2*R*,3*R*)-2,3-butanediol, chiral alcohols such as (*R*)-1-phenyl-1,2-ethanediol have served as valuable intermediates for synthesis of biologically or pharmacologically active compounds [42,43]. In addition, the NADH regeneration system catalyzed by formate dehydrogenase was confirmed to be effective since the asymmetric reduction of diacetyl led to production of 21.4 mM (*R*)-acetoin and 20.5 mM (2*R*,3*R*)-2,3-butanediol from 60 mM diacetyl with the use of only 0.25 mM NAD^+^. In total, ReBDH could serve as a potential versatile biocatalyst for the synthesis of (*R*)-stereospecific chiral alcohols.
